# Neuroscience of animal consciousness: still agnostic after all

**DOI:** 10.3389/fpsyg.2024.1456403

**Published:** 2024-10-09

**Authors:** Yoram Gutfreund

**Affiliations:** Department of Neurobiology, Rappaport Research Institute and Faculty of Medicine, Technion, Haifa, Israel

**Keywords:** animal consciousness, hard problem, animal cognition, neuroscience, distribution question

## Introduction

The question of animal consciousness, also known as the distribution question (Niikawa, 2020), is the question of which animal species share with us humans the enigmatic capability for conscious awareness. This is a philosophical question (Nagel, 1980) that stems from the “other minds problem” (Harnad, 2016). The implications of this question may influence ethical considerations and policy-making in human-animal interactions (Yeates, 2022) as well as challenges in diagnosing human consciousness in cases like locked-in syndrome (Bayne et al., 2024), and questions about machine consciousness (Schneider, 2020). Despite its importance and long-history of research, the distribution question is still highly debated among biologists and philosophers. The main problem is that consciousness in other animals cannot be directly observed, only inferred. Determining whether it is possible to scientifically infer consciousness in non-human animals, and how, is not trivial, with strong philosophical and scientific reasons to support an agnostic stance, implying that the question is unresolvable through scientific methods (Dawkins, 2017; Gutfreund, 2017, 2018; Hampton, 2021; Roige, 2023). However, objections to the agnostic stance are also common (Birch et al., 2022), and a recent consortium of animal scientists and philosophers endorsed the “New-York declaration on animal consciousness” (Andrews et al., 2024) which asserts, contrary to the agnostic stance, robust scientific evidence supporting the attribution of conscious experiences to other mammals and birds. The declaration also suggests a plausible likelihood of conscious experience across all vertebrates, including reptiles, amphibians, and fishes, as well as in many invertebrates, such as cephalopod mollusks, decapod crustaceans, and insects.

In this opinion paper, I critically assess the scientific claims for conscious experience in non-human animals, by reviewing two seminal studies published in leading journals (Nieder et al., 2020; Ben-Haim et al., 2021). These studies propose evidence of consciousness through observed neural activity and behavioral responses in non-human animals. The first, published in Science, argues for sensory consciousness in crows based on neural activity reflecting internal decisions. The second, published in PNAS, delineates perceptual awareness in rhesus monkeys, by exposing behavioral responses that are akin to subliminal versus conscious perceptions in humans. These studies exemplify the primary means for inferring animal consciousness: (1) identifying neural structures and activities akin to human neural correlates of consciousness (NCC) (Seth et al., 2005), and (2) recognizing behaviors in animals that resemble conscious behaviors in humans (Zlomuzica and Dere, 2022). I review the approaches and arguments presented in both studies and conclude that, while they provide novel, solid and general insights on animal cognition, the studies fall short of distinguishing cognitive abilities accompanied by conscious awareness from those that are not. Consequently, I argue that consciousness in non-human animals remains a subject of belief, beyond the reach of scientific validation.

## A neural correlate of sensory consciousness in a corvid bird

The authors of this paper (Nieder et al., 2020) define sensory consciousness as “the ability to have subjective experience that can be explicitly accessed and thus reported.” They continue by convincingly showing that the activity of neurons in the nidopallium caudolaterale (NCL) predicts the crow's behavioral choices in a delayed detection task at near-threshold stimulus, where the crows occasionally fail to detect a present visual stimulus or mistakenly detect a stimulus when it is absent. They then argue that “a difference between the neuronal activities of one reported perceptual state vs. the other for equal visual stimuli is considered to be a neural correlate of visual consciousness”. Hence, they conclude that the neural activity in NCL is correlated with subjective experiences (consciousness) of the crow. This line of reasoning has several problems. The first is that the crows do not report their subjective experiences, rather they make a behavioral decision as whether to respond according to a stimulus in sight or no stimulus in sight. This is commonly called a perceptual decision. Indeed, detection tasks like the one used in this paper have been used in primates and other species to study the neural correlates of perceptual decisions (Costello et al., 2016; Kwon et al., 2016). The subjective experience of the crow is hidden from us (Dennett, 1995; Staddon, 2000; Dawkins, 2017; Gutfreund, 2017; Hampton, 2021). Hence, a perceptual decision without a felt subjective experience (David et al., 2011) is a possibility that is equally consistent with the data and cannot be disregarded. Second, their argument that brain activity that changes systematically with the subject's report of whether it had perceived the stimulus is a testament of neural correlate of consciousness is problematic because the essence of all perceptual decisions is choosing one way or the other given the same sensory input (Gold and Shadlen, 2007). Therefore, it remains unclear how the authors of the paper can distinguish neural correlates of perceptual decision from neural correlates of consciousness (NCC), and what, in their findings, is different from similar findings in other species that did not provoke claims of NCC (Horwitz et al., 2004; Kwon et al., 2016). The third problem is that if their assumption about NCC is true, all active organisms' behaviors should be indicative of consciousness, as all animals make choices that are determined by the integration of noisy sensory evidence with internal states (Gordus et al., 2015) and a behavioral choice must be represented somehow in the neural activity of the brain.

An intriguing aspect of the experiment is that the crows were trained to respond conditionally, depending on the color of a cue given after the delay period (rule-based response). The ability to learn a rule-based task requires behavioral flexibility (intelligence) that not all animals possess (Maes et al., 2015). The rule-based behavior dissociates between the motor response and the perceptual decision. Thus, it enables the experimenter to isolate neural correlates of perceptual decision, as was done brilliantly in the target paper. But perception does not equal sensory consciousness. The former is the detection of the stimulus, whereas the latter is the subjective *experience*/*feeling* of the stimulus (awareness). The results show that neurons in the NCL of the crow code an internal representation that is used to control stimulus-dependent behaviors. Identifying such coding in the brain of the crow is an important achievement. However, the extra-step of inferring sensory consciousness is not a direct outcome of the evidence.

In the Discussion section, the authors refer to the distinction made sometimes in the philosophical literature between phenomenal consciousness (P-consciousness, the feeling or experience itself) and access consciousness (A-consciousness, the report or use of the internal experience) (Block, 2002). They admit that it is unclear whether crows have P-consciousness. However, it only makes sense to use the term A-consciousness if it is accompanied by P-consciousness, otherwise all animals and other adaptively behaving systems can be said to have A-consciousness (Naccache, 2018). Therefore, access and use of internal representations alone cannot serve as a marker for sensory consciousness.

## Disentangling perceptual awareness from non-conscious processing in rhesus monkeys (*Macaca mulatta*)

The authors of this paper (Ben-Haim et al., 2021) adopt a purely behavioral approach to propose an empirical test for consciousness in non-verbal animals. They examine the eye movements of monkeys engaged in a cueing paradigm. In this simple paradigm, the monkey must shift its gaze to a circular target on the screen to earn a reward. The target appears randomly on either the right or left side of the screen. Just before the target's appearance, a square-shaped visual cue is briefly shown on the opposite side of the forthcoming target. Notably, the cue duration varies: in some trials, it lasts just 17 ms, while in others it extends to 400 ms. The monkeys quickly learn to use the longer cues to predict where the target will appear, as indicated by significantly faster reaction times in these trials compared to those without a cue. Because the target side is always opposite from the cue side this learning highlights a cognitive ability to dissociate between the cue location and the target location. Crucially, in trials when the cue is briefly flashed, the monkeys do not make this dissociation and instead reflexively shift their attention to the cue's location, resulting in longer reaction times to the target located on the opposite side. The authors argue that the distinct behavioral responses to the salient (long) vs. non-salient (short) cues suggest the presence of conscious vs. non-conscious perception of the cue. To support this claim the authors show that humans performing the same task report being aware of the cue (conscious perception) when it enables the dissociation between the cue location and the target location (I see the cue, I anticipate the target to appear on the opposite side) and report not being aware of the briefly presented cue even though it induces the same reflexive response as in the monkey (subliminal response).

Paradoxically, this reliance on the subjective report of humans to support that the test is valid for discrimination between conscious and non-conscious perception in non-human animals, is the major drawback. Showing that an animal responds like a human only brings us back to the original distribution question that we started with: does an animal that responds like a human also feels like a human? The human-based argument for the presence of consciousness in animals is therefore circular and the behavioral test by no means answers the question.

The test developed by Ben-Haim and colleagues is intriguing as it contrasts learned responses with innate ones. Innate responses typically involve directing attention toward the location of a sudden, flashing stimulus. However, the findings indicate that monkeys, humans, and likely other animals can learn to override these innate responses with learned behaviors that are contrary to them. For an animal to learn and produce such counter-responses, the stimulus must be sufficiently salient and noticeable (Prasad and Mishra, 2019). This aligns with established theories that salient stimuli can enter working memory and engage higher cognitive processes, which can, if needed, inhibit innate reflexes (Diamond, 2013). In humans such salient stimuli trigger conscious awareness. How can we tell if the non-verbal animal is also aware of the stimulus? The possibility of learned responses without awareness of the stimulus is equally consistent with the observations.

## Summary

The papers discussed in this article focus on specific experiments and animal species (crows and macaques), yet they share a common assumption that human-like cognitive abilities or responses in animals indicate consciousness. This notion is not unique within the scientific literature that suggests the demonstration of consciousness in animals (e.g., Barron and Klein, 2016; Bronfman et al., 2016; Butler and Cotterill, 2006; Feinberg and Mallatt, 2016). A common theme across the studies is the merging of consciousness—defined as the private experiences and feelings of the subject—with cognition, which includes covert behaviors mediated by the brain such as perception, planning, decision-making, attention, and learning. Therefore, the main criticism, illustrated above, that the observed evidence fails to distinguish between felt experiences from cognitive behaviors without felt experiences, can be generalized across a broad spectrum of studies about animal consciousness. I believe that the common bias toward favoring the hypothesis that includes felt experiences stems from anthropomorphism—the tendency to believe that animals or systems that resemble us also experience feelings similar to ours (Varella, 2018). While anthropomorphism is natural, sensible, and important for guiding decisions about animal welfare, it cannot replace rigorous scientific reasoning. Therefore, I maintain an agnostic stance, arguing that the limitations of the above papers in inferring consciousness in animals is not a problem of premature science but a fundamental, unsolvable problem. We neuroscientists are unable to provide answers that transcend our personal beliefs because we can only observe cognitive behaviors and/or their underlying neural mechanisms, and the relationship between cognition and consciousness remains elusive.
